# Applying regulatory science in traditional chinese medicines for improving public safety and facilitating innovation in China: a scoping review and regulatory implications

**DOI:** 10.1186/s13020-021-00433-2

**Published:** 2021-02-16

**Authors:** Zuanji Liang, Yunfeng Lai, Meng Li, Junnan Shi, Chi Ieong Lei, Hao Hu, Carolina Oi Lam Ung

**Affiliations:** State Key Laboratory of Quality Research in Chinese Medicine, Institute of Chinese Medical Science, University of Macau, Macao Taipa, China

**Keywords:** Regulatory Science, China, Traditional medicine, TCM, Drug Regulatory Authority, National Medical Products Administration

## Abstract

**Background:**

The National Medical Products Administration (NMPA) in China has set to advance the regulatory capacity of traditional Chinese medicines (TCMs) with the adoption of regulatory science (RS). However, the priority of actions at the interface of RS and TCMs were yet to be defined. This research aims to identify the priority areas and summarize core actions for advancing RS for traditional medicines in China.

**Methods:**

A mixed approach of documentary analysis of government policies, regulations and official information about TCMs regulation in China, and a scoping review of literature using 4 databases (PubMed, ScienceDirect, Scopus and CNKI) on major concerns in TCMs regulation was employed.

**Results:**

Ten priority areas in the development of TCM-related regulatory science in China have been identified, including: (1) modernizing the regulatory system with a holistic approach; (2) advancing the methodology for the quality control of TCMs; (3) fostering the control mechanism of TCMs manufacturing process; (4) improving clinical evaluation of TCMs and leveraging real world data; (5) re-evaluation of TCMs injection; (6) developing evaluation standards for classic TCMs formula; (7) harnessing diverse data to improve pharmacovigilance of TCMs; (8) evaluating the value of integrative medicine in clinical practice with scientific research; (9) advancing the regulatory capacity to encourage innovation in TCMs; and (10) advancing a vision of collaboration for RS development in TCMs.

**Conclusions:**

RS for TCMs in China encompasses revolution of operational procedures, advancement in science and technology, and cross-disciplinary collaborations. Such experiences could be integrated in the communications among drug regulatory authorities to promote standardized and scientific regulation of traditional medicines.

## Background

Regulatory Science (RS) is a comparatively new discipline that has been adopted by drug regulatory authorities (DRAs) with the objectives to enhance the scientific rationale supporting their benefit/risk analysis and regulatory decisions based on best available science [1]. The Food and Drug Administration (FDA) in the US considers RS as a science of developing new tools, standards, and approaches to assess the safety, efficacy, quality, and performance of all FDA-regulated products [2]. Similarly, the European Medicines Agency (EMA) in the European Union defines RS as a range of scientific disciplines that are applied to the quality, safety and efficacy assessment of medicinal products and emphasizes on the entirety of the product lifecycle [3]. RS to the Pharmaceuticals and Medical Devices Agency (PMDA) in Japan plays an important role in optimally adapting technology achievements to social and human needs by making precise prediction, evaluation and judgment based on the best evidence available [4]. RS has been increasingly considered as the scientific discipline underpinning the scientific and technical foundations of drug regulatory authorities (DRAs) across the countries.

In China, the National Medical Products Administration (NMPA) highly recognized the values of RS to form the basis of regulatory activities. In May 2019, the NMPA launched the Action Plan of Regulatory Science which is the first official initiative that synchronizes the global trend of RS development [5]. To enhance the regulatory capacity, it is clearly stated in the Action Plan that the regulatory agency is committed to developing new regulatory tools, standards and methods in responses to both experienced and foreseeable challenges from the practical situation. The overall goal is for the regulatory decision-making process at the NMPA to become more scientific, forward-looking and adaptable in the new era of regulatory paradigm. According to the Deputy Director of NMPA, “*By developing and adopting regulatory science, the drug regulation system in China will adhere to the five main attributes of innovation, quality, efficiency, system, and capability* [6].”

The Action Plan of RS listed out the first batch of 9 key RS development areas in China which broadly covered a wide range of regulated products (such as pharmaceutical products, medical device, and drug-device combination products) and technology (such as cell and gene therapy, nanotechnology, and real world evidence) [5]. One of the 9 key RS areas was specifically dedicated to the evaluation of the safety of traditional Chinese medicines (TCMs) informed with the clinical practice of Traditional Chinese Medicine (TCM) theory. TCM refers to medicinal substances and preparations used under the guidance of TCM theory, which contains herbs, minerals and animal substances. This has successfully marked the unique application of RS on traditional medicine calling for immediate actions to accelerate advancement. While the RS plan in China has emphasized the importance of TCMs, further specifications about the areas of concerns have not been systematically analyzed. This paper aims to present a snapshot about applying RS in the regulation of TCMs in China and to propose actions essential to the support such development in the best interest of public health and industry growth. It is envisioned that the experiences of developing RS in TCMs in China may also become a valuable RS development blueprint for other DRAs which strive to ensure the quality use of traditional and complementary medicines.

## Methods

A mixed approach involving documentary analysis of government policies, regulations and official information about TCMs and RS in China, as well as relevant a scoping review of literature was employed in this study since Jan 01, 2000 (3 years before the introduction of the concept about developing scientific approaches to improve the health of the population in China [7]) to Jul 31, 2020. The publicly government documents were identified from the official websites: The State Council, The People’s Republic of China (http://english.www.gov.cn/), The National Medical Products Administration (http://english.nmpa.gov.cn/), and The National Administration of Traditional Chinese Medicine (http://www.satcm.gov.cn/), The Center for Drug Evaluation of NMPA (http://www.cde.org.cn/), and The Center for Information of NMPA (http://www.sfdaic.org.cn). The government collected documents were classified and further analyzed according to the registration category, market access requirements and post-marketing regulation of TCM.

On the other hand, literature published since 2000 was searched from 3 English electronic databases (including PubMed, ScienceDirect, Scopus) and 1 Chinese electronic database (Chinese National Knowledge Infrastructure, CNKI) using the search terms: (“regulatory science” OR “government regulation” OR “Government Regulation” [MeSH]) AND (“traditional Chinese medicine*” OR “TCM” OR “traditional medicine*” OR “integrative Medicine” OR “Medicine, Traditional” [MeSH] OR “Medicine, Chinese Traditional” [MeSH] OR “Integrative Medicine” [MeSH]) AND “China”. Publications which refer to the study, review, comment of TCMs regulation were selected independently by 2 researchers and included in this study for further data analysis upon careful assessment and agreement by the research team. Studies which were about clinical trial, meta-analysis, or randomized controlled trials of TCMs were excluded.

## Results

### Ten priority areas of RS development in TCMs

The core responsibility for the NMPA is to ensure the safety, effectiveness and quality control data of TCMs (and all the pharmaceutical products) approved for marketing. For achieving such goals, strengthening the regulatory systems through developing RS is considered a core action [8, 9]. At present, the TCMs regulated by NMPA are divided into medicinal materials, decoction pieces and finished medicines. Considering the “complexity” and “variability” of the processes of “from planting and production to final clinical application”, and “from medicinal materials and decoction pieces to finished products”, the regulatory requirements for TCMs is distinctively different from that for chemical drugs [10]. Advancing RS by the NMPA will have important implications for the regulatory capacity to overcome challenges in the decision-making process about TCMs. In the following, 10 priorities areas in TCMs regulation worth the leading effort of RS development by the NMPA are proposed.

#### Priority Area 1: modernizing the regulatory system with a holistic approach

The regulation of TCMs is a prolonged and highly complex process starting from the planting field to bedside and beyond. Each domain of the process requires different yet standardized and continuous skillset at technical and operational levels. However, the current drug regulatory system in China is characterized by a tiered approach involving the central and the local governance over various phases of regulatory process. At different stages of the drug lifecycle, the regulation process is generally carried out by different offices of national and provincial drug regulatory departments [11]. In other words, the process of drug regulation is divided into several operation blocks such as registration, production, circulation and adverse reaction monitoring [11, 12]. Besides, under the phased mode of the regulatory process, each regulatory department has its own scope of regulation, and an apparent lack of effective communication between different regulatory departments, as well as between each regulatory authority and the applicants [12]. This may inevitably affect the availability and accessibility of information necessary for regulatory decision-making, and loopholes in the overall regulatory paradigm may thus result, affecting the rigor of the regulatory mechanism [13]. At the same time, the efficiency in the use of regulatory resources may also be jeopardized.

From the experiences of DRAs, a shift towards a holistic approach that integrates product-base system-base approaches is recommended to ensure drug safety and risk management throughout the product lifecycle. As such, the operation blocks at each stage of product lifecycle can be connected with data flow and information transfer to complete the regulatory chain that allows a helicopter view of planning, monitoring and control achieving dynamic management. In the newly revised Drug Administration Law and Provisions for Drug Registration, the concept of marketing authorization holder (MAH) has been officially adopted. On one hand, it allows domestic research institutions, drug manufacturers, and individuals to hold licenses to market drug products without holding a manufacturing license for a facility. On the other hand, more importantly, it sets forth a concrete, consolidated list of MAH responsibilities. Together with stronger pharmacovigilance and post-market surveillance system in place, MAH is now required to take responsibility of the safety, efficacy, and quality of their drugs during the entire “lifecycle,” including non-clinical research, clinical trials, manufacture, distribution, and post-marketing surveillance. This echoes the regulatory requirements set forth by the DRAs in the US, EU and Japan [14]. In addition, NMPA has also adopted the International Council for Harmonization of Technical Requirements for Pharmaceuticals for Human Use (ICH) Q12 “Technical and regulatory considerations for pharmaceutical product lifecycle management” that emphasizes on the importance of data availability and stability to inform the regulatory decisions made throughout the product lifecycle [15, 16].

Stable and controllable quality of TCMs is the basis of stable quality and the curative effect of TCMs. For this, NMPA issued the Good Agricultural Practice (GAP) in 2002 [17] in order to help minimize the risks of germplasm confusion, as well as the overuse of pesticide and fertilizer in the growing of medicinal materials. On the other hand, WHO guidelines on good agricultural and collection practices (‎GACP) [18]‎ *for medicinal plants were published in 2003. Another area of concern regarding TCMs was the possible use of endangered animals. As early as 1987, the State Council of China issued the Regulations on the Protection and Management of Wild Medicinal Materials Resources [19] aiming to protect wildlife resources and discourage artificial breeding with legal forces.

In order to meet the requirements of traceability and monitoring of the quality of TCM products, the Opinions of the State Council on Accelerating the Construction of Traceability System for Important Products [20] was implemented followed by the Guiding Opinions on the Construction of Drug Information Traceability Information System [21], Guidelines for drug Traceability Information System Construction [22] and Encoding Requirements for Drug Traceability Code [23]. Collectively, these documents provide a framework of standard requirements for developing drug traceability system to ensure the traceability of a drug’s entire lifecycle. Meanwhile, NMPA also launched the Action Plan for Accelerating the Smart Regulation of Drugs [24], which encourages the implementation of a big data regulatory infrastructure based on the drug traceability system to further improve the drug surveillance system.

The core responsibility of developing RS here is to advance and improve the existing regulatory systems and infrastructure to reinforce the monitoring and traceability of TCMs across the entire product lifecycle from raw materials to bedside [25]. Actions are needed to strengthen the inter-departmental regulatory coordination mechanism and encourage internal and external communication and collaboration to engage diverse key stakeholders such as the Centers for Disease Control, MAH, manufacturer, distributors, users, and other technical institutions. Information technology and other innovations are needed to lead the development of scientific infrastructure for the regulatory agencies at different levels to advance the data collection and sharing, and surveillance of TCMs throughout their lifecycle.

#### Priority Area 2: advancing the methodology for the quality control of TCMs

At present, the scientific and systematic research on medicinal materials, decoction pieces and extracts are still ongoing. In the Chinese Pharmacopoeia (Ch.P) 2015, out of the 655 enlisted medicinal materials, decoction pieces and extracts, 210 lacked the qualitative and quantitative analysis of the major constituents while the systematic research for more than 80 % of TCMs are yet to complete [26]. Even for those monographs and standards already developed (which already specify definitions, characters, identification, tests, assays, storage information, and more), such standards may not be specific enough and sometimes fall short to differentiate highly similar herbal species. Considering the various levels of complexity in the combination of the ingredients and the multiple chemical constituents of each ingredient, as well as the lack of standardization in the formulation when used in practical setting, the quality and standard data currently available is not sufficient to support the formulation of a comprehensive and effective system of quality standards for TCMs [26].

The efficacy and safety of TCMs are highly associated with the quality. However, unlike chemical synthetic drugs, TCMs is a complex chemical system and the quality is attributed by the chemical components of many structurally diverse compounds. Not surprisingly, the development of quality control and standards for most herbal materials, processed herbal preparation, and the final product of TCMs are highly challenging [10]. Moreover, the characteristics of synergistic reactions among different components, action channels and effect targets are difficult to be elucidated by the study model of “single component and single target” in modern medicine. Therefore, a scientific and effective system for evaluating the efficacy and safety of TCMs should be prioritized on the quest.

Great progress has been made in recent years to clarify the substance base and the mechanisms of actions for some TCMs. For the development of quality standards, biological assay has been recommended to better ensure the quality of TCM sources, authentication of species and preparations, stability of intermediate extracts, and the consistency of preparations [27]. The Guiding Principles for Bioassay of TCMs [28] has been adopted and published in General Principles of Ch.P for quality control of TCM. Similarly, the Botanical Drug Development Guidance for Industry also clearly indicates that biological assay is an important content of new drug registration review of botanicals in the US [29]. In addition, the concept of quality markers [30, 31], biological activity and biological response [32], as well as intelligent quality management [33] have also been suggested for the evaluation of TCMs quality [34–37]. Meanwhile, modern analytical technologies such as mass spectrum and chemometrics hold promising potentials to continuously contribute to the identification of potential quality markers of the herbs [38, 39], and other TCMs preparations [40, 41].

With the development of RS, it is urgent to develop new technologies and methods for advancing the approaches in the quality standards, control and evaluation, as well as the traceability system that addresses the unique features of herbs and TCMs across the entire lifecycle. Recently, the National Natural Science Foundation of China listed the quality markers of TCMs as a key project in 2018. Academic experts have already conducted discussions about the applicability of the quality markers of TCMs and published hundreds of related articles. Some key universities and large-scale Chinese medicine enterprises in China have already carried out relevant scientific research to be integrated in the drug development process. Developing RS should benefit not only the development of new methods and technologies, but also the coordination of research patterns and research efforts to justify the rationale for any developments in TCMs quality control and assessment system in a more efficient manner.

#### Priority Area 3: fostering the control mechanism of TCMs manufacturing process

Currently, the quality control through sample testing of raw materials, intermediate products and final products are mostly conducted manually in a detached manner. As such, the test results may not be able to fully and comprehensively reflect the quality of TCMs, and the approach is neither conducive in detecting changes in the production process and nor sensitive in identifying problems in time [42]. This is especially the case for traditional pharmaceutical process of TCMs that features the following: (1) low level of automation that affects the consistency of the operation and thus the product quality; (2) poor information integration capability that supports an automatic control system; (3) insufficient openness of the system that affects the interoperability and interchangeability of the bottom control appliances; and (4) poor maintainability and unreliable dependability of the appliance [43]. An operational procedure that strictly and accurately control the various control points (pretreatment, extraction, separation, concentration, dehydration and formulation) of the TCMs pharmaceutical process leading to a technological process control will have a direct impact on quality and effect of products.

In the US, the FDA launched the process analysis technology (PAT) program in 2002 and issued the Guidance for Industry about PAT in 2004 [44]. Gradually, PAT became the reform direction and research focus in the field of quality control in pharmaceutical industry. In 2016, the Ministry of Industry and Information Technology in China launched the “Planning Guide for the Development of Pharmaceutical Industry” which proposed the use of PAT to optimize pharmaceutical process and quality control, and realize the technical convergence and product quality consistency of pharmaceutical products [45]. As a powerful PAT tool, near-infrared spectroscopy has been adopted in real-time monitoring of TCMs technological processes [46]. Similarly, the NMPA also encourages the use of Quality by Design (QbD) as a means to continuously improve the consistency of the product quality and to monitor for contamination or other production failures during the manufacturing process. The concept of QbD has evolved with the issuance of ICH Q8 (R2) (Pharmaceutical Development), ICH Q9 (Quality Risk Management), and ICH Q10 (Pharmaceutical Quality System) [47]. For drug regulators, analysis of QbD information would help acquire a better understanding about the possible variations, grasp the inevitable differences between batches, and ensure that the review conclusions are as scientific as possible.

The use of novel science and technologies, such as analytical technology to monitor and control processes or statistical methods to discover any variations in manufacturing process or product quality, is vital in light of the complexity of manufacturing process and innovation of TCMs. To this end, it is important for the NMPA to reinforce the research capacity guided by RS to assess how to develop and best use new technologies to the assurance of product safety, efficacy, and quality, and to do so in collaboration with industry and academia when developing regulatory policies corresponding to these innovations.

#### Priority Area 4: improving clinical evaluation of TCMs and leveraging real world data

The mainstream of medical knowledge and practice in the world is evidence-based medicine which recognizes the values of scientific evidence, and the efficacy and safety of any clinical interventions is primarily determined by clinical evidence. Undoubtedly, it is an important task to leverage evidence-based clinical study and evaluation of TCMs [48]. Nevertheless, it remains highly challenging to integrate the TCMs characteristics into the design methods for various reasons. Firstly, the design quality of randomized controlled trials (RCTs) is often weak at the randomization and allocation method and the evaluation of efficacy outcome in the blind trials. Secondly, while the diagnosis based on the TCM theory is an important factor in identifying eligible research objects in clinical research of TCMs, there is a lack of internationally recognized diagnostic criteria. Thirdly, there is a lack of standardized and quantitative evaluation indicators of curative effects such as “treating the root cause” or “strengthening the foundation” affecting the evaluation of clinical efficacy of TCMs. An additional challenge in TCMs research is the lack of standardization in the products, which is further complicated by the features of multiple active components found in numerous chemical compounds commonly seen in TCMs.

Evidence-based clinical practice guidelines of TCM and modified research models of the randomized controlled trial (RCT) have seen much progress in recent years. For instance, N of 1 trial, pragmatic trials, add-on design, expertise-based trials have been used for assessing clinical effect of TCM interventions including TCMs and other TCM practice [49]. Explanatory RCT has been used to test the efficacy in a research setting using highly selected study subjects and under highly controlled conditions [50]. Such design optimally balances out the effect of any actual or potential confounding factors when evaluating the efficacy of TCMs in comparison to placebo or active drugs. The pragmatic RCT design is more closely related to the “real world” clinical settings and is often used in the study of effectiveness comparison [51]. A series of one (N-of-one) trial is a special type of crossover trial which involves rounds of treatment crossovers within a single subject. This research design may be further repeated several times to confirm the results about the effectiveness of the TCMs in question [52]. To develop evidence about TCMs, the National Administration of Traditional Chinese Medicine established the Evidence-based Medicine Center of TCM in collaboration with the Chinese Academy of Chinese Medical Sciences in 2019. Around the same period, the “Evidence-based Capacity Building Project of TCM” [53] was also launched to promote the capacity building of developing evidence base for TCMs at clinical research bases and treatment centers in 31 provinces in China.

Furthermore, with the advent of the big data era, evidence-based evaluation methods of TCMs need to be continuously developed and updated. In China, the importance of real world evidence was to fill the research gaps due to the limitations of traditional TCMs clinical trials and to address the needs for additional evidence to inform policy decisions [54]. NMPA recently published the “Guiding Principles of Real World Evidence Supporting Drug Research and development and Evaluation (Trial)” [55], which proposed innovative strategies of combining real world study with RCT as new paths of clinical research and development, and clinical effectiveness and safety evaluation, thus supporting drug regulatory decision-making. Collectively, it requires both modern clinical trial design and statistically sound methods to leverage clinical data and real world evidence for the evidence-based approach in the evaluation of TCMs treatment.

The value of RS here is to encourage encouraging innovation in clinical evaluation through collaborating with other stakeholders to develop and modernize clinical trial designs and statistical methods to encourage innovation in clinical evaluation of TCMs. At the same time, the NMPA should reinforce the regulations about the confidentiality, privacy protection, data sharing governance, and ethical approval of data collected from various sources. For this, the adoption of RS should see the outcome of a systematic approach in data collection and data quality control through the development of policy framework, regulatory documents and technical guidance. This should be a joint effort across institutions and stakeholders to foster mutual understanding and cooperation so that common goals and agendas can be shared among researchers, practitioners, and policymakers. As such, developing RS in this area will help ensure for the development and application of high-quality real world evidence to policy and regulatory decisions by the NMPA.

#### Priority area 5: re‐evaluation of TCM injection

A TCM injection is a sterile preparation that may contain one or more purified extract of herbal materials formulated as a solution, emulsion, powder, or concentrated solution for injection into human body [56]. They are a unique type of TCMs preparation that has been widely used in clinical practice in China and remain the most popular dosage forms of TCMs due to their remarkable effects in certain diseases [57, 58] accounting for one third of all TCMs sales in hospitals [59]. However, there are continuous concerns over the safety of the TCM injections in light of the significant number of adverse drug reaction (ADR) reports [60–62]. Statistics show that ADR reports related to TCM injections account for > 50% of total ADR reports related to TCMs [63] resulting in a series of public warnings and other regulatory actions by the NMPA since 2006 [64].

Safety concerns over TCM injections include allergic reactions to any of the components or may be related to the insufficient quality control of the preparations which may be attributed to insufficient basic research and poor controllability of quality standards. According to the study by Li et al. [65], among the 134 of TCM injections manufactured by 224 manufacturers approved for sale by the NMPA in 2017, only 5 of them had their monographs published in the Ch.P (2015). The quality standards of the remaining 129 TCM injections (92.3%) were documented in other national guidelines which set a comparatively lower benchmark of quality or consistency and may not undergo regular review and update as the monographs in Ch.P. The substandard requirements for the quality control of TCM injections have profound implications for the effective control of product quality [63]. This is especially the case for the TCM injections which were approved for sale many years ago and were thus exempted from the regulatory requirements about the safety and efficacy of the current standards.

There is an urgent need for post-marketing re-evaluation of TCM injections by methodological and rigorous testing that emphasizes on quality controllability and risk evaluation and management [66, 67]. In 2008, the Ministry of Health and NMPA and the National Administration of Traditional Chinese Medicine jointly issued the “Basic Principles for Clinical Use for TCM Injections” [68]. In 2009, the NMPA also issued the Work Plan for Re-evaluation of the Safety of TCM Injections [69] which called for comprehensive actions in risk investigation in production and quality control, risk-benefit evaluation across batches, and improvement of product quality standards. Seven guidance documents were subsequently published for public reflection in 2010 on safety evaluation of TCM injections in non-clinical research, clinical research, production process, quality controllability, risk control capability evaluation, risk benefit evaluation and risk management plan [70]. In 2015, “Opinions on Deepening the Reform of Review and Approval System and Encouraging the Innovation of Pharmaceuticals and Medical Devices” [71] re-emphasized the re-evaluation of pharmaceutical injections.

To this end, the development of RS should focus on developing a platform for safety evaluation that is compatible with TCM toxicity attributes to effectively address specific TCM toxicities and safety concerns [72, 73]. Network pharmacology which considers multi-target strategies over single-target approaches may offer an alternative and modernized way to investigate the ADR and toxicity of drugs [74, 75]. The application of drug toxicology genomics and/or metabolomics at the early-stage of TCMs toxicity investigation should be explored [76–78]. Other techniques such as in-vitro multi-parameter cell-based imaging method that featured high throughput may also be used as an alternative to animal testing to develop a mechanistic understanding of TCMs ADR. The post-marketing clinical research also provides important information needed for the re-evaluation of TCM injections. Using data from the pre-existing hospital information systems, re-evaluation studies of TCM injections about the post-marketing safety can be carried out using prospective nested case-control and prescription sequence analysis designs [79–81]. Other cutting-edge technologies such as fast screening methods based on drug-metabolizing enzymes and receptor pathways, computational toxicology, and molecular toxicology may offer a new system of TCMs safety evaluation techniques [82, 83].

#### Priority Area 6: developing evaluation standards for classic TCM formula

Ever since the National Administration of Traditional Chinese Medicine published the list of the first batch of classic TCM formula (CTCMF) in 2018 [84], CTCMF has become one of the most popular topics in the drug regulation sector in China. CTCMF refers to TCMs prescriptions passed down from the clinical practice of famous doctors in past dynasties which had been documented in ancient TCM textbooks, are still widely used, and have definite and distinctive curative effects which is based on experience of traditional use not necessarily contemporary RCTs. Under the assumption that the safety and efficacy of CTCMF have been verified through clinical practice for hundreds or thousands of years, the TCM Law [85] stipulates that, for drug registration purposes, the application will only need to include non-clinical safety data for the preparations of CTCMF. To further specify the registration requirements, the NMPA published three technical documents [86, 87], such as Administrative Provisions on Simplified Registration and Approval of CTCMF (Draft for Soliciting Opinions), to clearly define the evidence requirements for human use experience and to introduce the relevance of real world study as a source of human use experience [88].

Real world study is an important research method to establish high-quality clinical evidence of TCM intervention, which can embody the connotation of TCM to the maximum extent [89]. However, it remains a great challenge for the simplified application of CTCMF to determine: (1) the authenticity of the origins and sources of the raw herbal materials; (2) the quality standards and the substance benchmark of CTCMF and CTCMF preparations; (3) the basic requirements and principles for the comparative study of CTCMF preparations (standard decoction and new preparations); (4) the standard processing method and the right dosage range of CTCMF preparations; (5) the quality traceability system from medicinal materials to preparations that embodies the whole process quality control concept [90].

Multiple issues to be resolved through adopting RS may include: (1) development of quality markers of CTCMF to ensure quality stability and consistency; in the genuineness of medicinal materials and product industrialization; (2) determination of the genuineness of medicinal materials that specifies the origin, habitat, harvesting time, storage and transportation process, processing, resource balance, production scale of the raw herbs; (3) standardization of the preparation and manufacturing process from substance basis and standard decoction to industrial preparation; (4) information transferability and traceability across the product lifecycle incorporated with modern process control theory and norms (such as good engineering practice, GEP); (5) application of Good Agriculture Practice (GAP) to ensure quality traceability system of medicinal materials which may involve artificial cultivation and breeding [90].

#### Priority Area 7: harnessing diverse data to improve pharmacovigilance of TCMs

Effective pharmacovigilance is a key to the development of guidelines which ensures safe, effective use of TCMs. According to the definition of World Health Organization (WHO), Pharmacovigilance (PV) refers to the science and activities related to the detection, evaluation, understanding and prevention of adverse reactions or any other drug-related problems [91]. Adverse drug events (ADE), including adverse drug reactions (ADRs), drug quality problems, and drug interactions, will have a great impact on patients’ health and significantly increase their medical costs [92–94]. An efficient PV system can not only monitor, evaluate and prevent adverse reactions or other related medical events but also be a key step to improve the drug regulatory system, clinical practice and public health plan [95].

Nevertheless, China’s PV system is still developing as the current ADRs monitoring and reporting system fall short to fully support the regulation of TCMs across the product lifecycle. According to the experiences of DRAs, the utilization of healthcare database has gradually increased, especially in Europe, the US and Asia in recent years. These medical databases have been widely used to support drug discovery, comparative efficacy and safety evaluation of marketed drugs [96–99]. The US FDA and other medical regulatory agencies, pharmaceutical industry and drug safety researchers have been exploring the application of big data in PV [100]. Sentinel is a post-marketing monitoring system developed by the US FDA in 2008. As of 2018, there are medical data of more than 300 million people [101]. In order to promote cooperative research through the establishment of a health database network, the EMA has been coordinating European Network of Centres for Pharmacoepidemiology and Pharmacovigilance (ENCePP) in recent ten years, which includes about 200 public institutions and contract research organizations to participate in activities related to drug epidemiology and PV. Up to now, monitoring drug safety through big data has proved to be cost-effective and can quickly determine the internal links related to ADRs.

In the perspective of RS, although the development of real world evidence is at an early stage in China, a number of important government initiatives are already underway. One such program is the China Hospital PV System, which was started in 2015 by the National Centre for ADRs Monitoring at the NMPA. This nationwide program was designed to identify safety signals proactively and to assist the analysis of the association between drug exposure and ADE. This system combined with the current drug safety monitoring system helps to reinforce the PV capacity in the country. The system collects data in electronic medical records from 300 hospitals and from healthcare claims to generate real world drug safety evidence for integrative analysis using drug risk analytics tools to support regulatory decision-making [102]. China is also actively developing intelligent information technology for TCMs regulation. The Research Institute of TCM regulation has set up a “Big Data Research Center for TCM”, relying on the “Database of National Center for ADRs Monitoring”, uniting with the “Internet++” platform of TCMs nationwide and other eight centers of the institute, based on emerging technologies such as big data, blockchain and artificial intelligence, focusing on two key points: data marking in the whole lifecycle of TCM and timely identification of medication safety risks.

#### Priority Area 8: evaluating the value of integrative medicine in clinical practice with scientific research

Although the term integrative medicine (IM) has been used frequently in different healthcare settings, there is not a standard definition for it [103]. In China, IM refers to the integration of TCM modality and conventional medicine modality as TCM is usually practiced alongside with conventional medicine especially in hospital settings [104]. This is of particular importance for complex diseases which may not be addressed by one medical system alone. As with conventional medicine, IM emphasizes the importance of an evidence-base for effectiveness especially in the international perspective [103, 105]. Although there are many successful examples of clinical practice integrated at therapeutic level, there are apparently a limited number of trials on IM and thus lack of evidence for IM which suggests that an IM approach may be beneficial for certain conditions [106–108].

To conduct trials on IM remains highly challenging due to the limitation of clinical trial designs [105]. Despite decades of research and integration, the fundamentals of TCM remain largely unchanged and its theories inexplicable to science [109–111]. The research evidence on the effectiveness of IM provided as a package of care is limited due to its complex nature and definition, lack of standardization and challenges in methodological design [112–114]. However, major breakthroughs have been reported recently. Since the COVID-19 outbreak in China, it is mandated that the use of IM must be strengthened to manage the pandemic at a national level. The scientific and technological emergency research project “Clinical Research on Prevention and Treatment of Pneumonia Infected by New Coronavirus by Combination of Chinese and Western Medicine” was officially launched [115]. As a result, real-world evidence for IM in the prevention and treatment of the infection has been mounting [116–119].

In order to improve the quality of IM research, supporting policies and initiatives are already in place. The Outline of the National Strategic Plan for the Development of TCM (2016–2030) mentioned that “using modern science and technology to promote the integration of Chinese and Western medicine, complementary advantages and collaborative innovation” is one of the key tasks [120]. Local governments, such as Guangdong Province, have issued 11 local standards, such as General Rules for the Revision of Clinical Practice Guidelines of TCM (Integrated Chinese and Western Medicine) [121], which provide methodological basis for the construction of clinical practice, comprehensive acquisition of evidence and evaluation. Methodology wise, pragmatic RCTs or observational studies with nested qualitative approaches (with both practitioners and patients) that mimic the complexity of health care provision in the ‘real world’ situations are worth investigating [103].

To combines two knowledge systems to become an alternative medical model, optimum evidence and optimum research methodology are essential [122]. Therefore, it is necessary to establish the curative effect and risk evaluation based on evidence-based medicine to support the integration of Chinese Medicine and conventional medicine. RS in IM may focus on developing reporting guidelines which provide guidance for researchers and reviewers to conduct and assess evidence about IM and to facilitate systematic reviews on effectiveness.

#### Priority Area 9: advancing the regulatory capacity to encourage innovation in TCMs

Chinese herbal medicines provide a wealth of potential source materials for drug discovery [123]. According to Deng, 3563 extracts and 5000 single compounds from 3000 therapeutic herbal medicines have been collected in China in 2007 [124]. A study in 2011 also revealed that in China, at least 130–140 new drugs, either single chemical entities extracted from herbal medicines or synthetically modified compounds, are currently in clinical use [123]. With the advantage of a long history of and a strong theory base for the clinical use, drug candidates have been successfully identified for drug discovery. For instance, goldthread (*Coptis chinensis* Franch.) which has been used as a TCMs to treat inflammatory symptoms and infectious conditions for more than 3000 years [125], was found to contain berberine. With modern science, it was found that berberine not only possesses antibiotic and anti-inflammatory properties [126, 127], but also exhibits beneficial effects for inflammatory bowel diseases, and modulates the activities of ERK, p38 MARK, and JNK to suppress T17 and T1 T cell differentiation suggesting the potentials to becoming a therapeutic agent to treat type 1 diabetes mellitus [128]. Further studies also suggested that berberine may also be a promising treatment for cancer, depression, hypertension, and hypercholesterolemia [129].

Biochemical techniques and cutting-edge technologies have made available new approaches in drug discovery from Chinese herbal medicines from the predrug stage and the quasi drug stage into the full-drug stage [130]. The virtual mapping between databases of Chinese herbal ingredients and molecular targets of diseases represents a new approach of drug discovery [123]. To search for an active herbal ingredient or lead compound from herbal materials, new technologies such as supercritical carbon dioxide extraction technology, membrane separation technology, semi-bionic extraction method, molecular distillation technology, and enzyme method in extracting effective components of medicinal plants have been developed [131–134]. Systems pharmacology uses both experiments and computational analysis of regulatory networks to develop a new understanding of drug action across multiple scales of complexity ranging from molecular and cellular levels to tissue and organism levels [135]. Genomic, proteomic and metabolomic technologies, network analysis, and other high-throughput studies, as well as structural and biochemical studies, are also being used to conduct systems-level analysis of drug action and molecular targets [136, 137]. With continuous development, nanotechnology has been applied to the research and development of TCMs particularly for formulation optimization such as the controlled preparation of TCMs using specific microstructure and surface properties [138, 139].

These novel and increasingly complex approaches to drug discovery from Chinese herbal medicines present growing challenges to NMPA’s readiness to evaluate new products. For this, RS must be one step ahead to equip the NMPA with the necessary tools and methods to reliably assess the safety and efficacy of products derived from these new scientific developments. New assessment tools and methodologies needed for the evaluation process of innovative products, and advancements in the expertise and infrastructure to evaluate new and emerging technologies can be facilitated through active collaboration with external partners. Reaching out to manufacturers and clinicians to identify areas of high public health need with few existing effective interventions might be of strategic advantages. Efforts should also be made to promote the development, standardization, and validation of new techniques for the assessment of quality, safety and effectiveness of new products across the sectors. Cross-disciplinary RS training and research to address scientific gaps and challenges posed by novel products are also needed.

#### Priority Area 10: advancing a vision of collaboration for RS development in TCMs

The challenge of drug innovation and development is not just a matter for drug regulatory authorities. It is necessary to study new science, new technology, new methods and new tools, to jointly promote and truly obtain the progress of applied science and technology, and to cooperate with all stakeholders. Established in 2013, China Society for Drug Regulation, which is headed by the NMPA, is a collaborative platform among government, academia and industry. At present, 17 professional committees have been set up to assist NMPA in the research about the theory, technology and method of drug regulation, and promote the innovation and development of drug RS in China.

Nevertheless, the technical support for RS development needs further strengthening. As such, NMPA has established multiple research bases and centers in universities for driving TCMs RS development such as Peking University and Tsinghua University [140–146], aiming to strengthen the construction of RS (Table 1). To consolidate the resources for RS development, NMPA has signed a series of cooperation agreements with universities and research institutes such as the Chinese Medicine Regulatory Scientific Research Cooperation Agreement with the Chinese Academy of Chinese Medical Sciences and Beijing University of Chinese Medicine to establish the Chinese Medicine RS Research Center (Research Institute) [142]. The overall goal is to resolve any technical challenges in TCMs evaluation and regulation through joint efforts in research on policies and mechanisms of TCMs innovation, as well as education and training scientific talents in TCMs regulation.

At the same time, NMPA has actively established different forms of strategic alliances with other countries, and strengthened cooperation and exchanges in its regulatory capacity and standard setting. Such efforts are important for the agency to grasp the most advanced scientific research achievements and regulatory experiences, carry out practical and effective transformation, and enhance regulatory capacity and standards. At present, China’s international cooperation in drug regulation includes: ICH, International Coalition of Medicines Regulatory Authorities (ICMRA), the Pharmaceutical Inspection Convention and Pharmaceutical Inspection Co-operation Scheme (PIC/S), the Global Coalition for Regulatory Science Research (GCRSR), International Medical Device Regulators Forum (IMDRF), WHO Good Regulatory Practices (GRP), etc.

**Table 1:** Partnerships formed by NMPA for the development of RS

	Time	Collaborators	Collaborating research base	Key research areas
1	2013/09	Tianjin Binhai Food and Drug Administration and Tianjin Institute of Pharmaceutical Research	Tianjin Binhai Center for Food and Drug Regulatory Science Research	Drug and food technology and policy
2	2015/08	Peking University	Peking University Asia Pacific Economic Cooperation Regulatory Sciences Center of Excellence	Education and training, RS research and innovation, international collaboration, RS Information Technology and Policy
3	2016/12	Fudan University and University of Copenhagen	Sino-Danish Regulatory Science Center	Education and training, trainee exchange and professionals training RS program
4	2018/04	Tsinghua university	Institute of Regulatory Science and Chinese Medicine Research Institute	TCMs modernization and RS research
5	2019/04	Sichuan University	Medical Device Regulatory Science Research Base	RS in medical device
6	2019/06	Beijing University of Chinese Medicine	Chinese Medicine Regulatory Science Research Institute	RS in TCM
7	2019/06	China Academy of Chinese Medical Sciences	Chinese medicine Regulatory Science Research Center	RS in TCM
8	2019/10	Shenyang Pharmaceutical university	Drug Regulatory Science Research Base	Course development, Education and training, Research in RS
9	2019/12	Shandong university	Drug Regulatory Science Research Base	RS in pharmaceutical products
10	2019/12	South China University of Technology	Medical Device Regulatory Science Research Base	RS in medical device
11	2020/04	Shanxi Medical products Administration and Xi’an Jiaotong University	Northwest Institute of Pharmaceutical Regulatory Science	RS research, Training and Education

## Discussion

In this review, 10 priority areas in the development of TCM-related regulatory science in China have been identified, the major concerns reviewed, and core actions suggested for advancing RS for TCMs in China. As presented in Table 2, it has been proposed that NMPA when developing RS in TCMs has the prime duties to formulate scientific norms around the major concerns over the complexity and variability of the whole lifecycle of TCM, formulate scientific operational procedures (priority area 1), encourage the advancement and use of scientific means and technology (priority area 2,3,4,5,6,7,8,9), and develop inter-disciplinary collaborations (priority area 10) to address the goals in the RS initiative. The overall goal of the RS initiative in China and the proposed priority areas in TCMs share commonalities with the priorities set forth by the FDA [2] and EMA RS [3] in their respective RS action plan: improve the efficiency of the evaluation and approval process, deliver safe new products to patients faster, and strengthen the regulatory capacity to improve regulatory performance and thus patient outcomes.

**Table 2:** 10 priority areas and core actions summarized for advancing RS in TCMs

	Priority area	Major concerns	Core actions proposed
1	Modernizing the regulatory system with a holistic approach	(1) Existing regulatory mode (2) Drug lifecycle management	(1) Innovative regulation mode of drug lifecycle (2) Establishment of drug information traceability information system (3) The Action Plan for Accelerating the Smart Regulation of Drugs
2	Advancing the methodology for the quality control of TCMs	(1) Basic research of quality control (2) Innovative quality control methods	(1) Guiding Principles for Bioassay of TCM (2) Identify and evaluate quality marker
3	Fostering the control mechanism of TCMs manufacturing process	(1) Possible variation of quality in product manufacturing process (2) Many control points in TCMs manufacturing process	(1) Develop a PAT tool such as near-infrared spectroscopy (2) Guidelines for QbD concept
4	Improving clinical evaluation of TCMs and leveraging real world data	The clinical evaluation of TCMs remains many challenging	(1) Establishment of TCMs evidence-based Center (2) Initiation of the Evidence-based Capacity Building Project of TCM (3) Guidelines for drug discovery and review supported by real world evidence
5	Re-evaluation of TCM injection	The risk of TCM injection safety	(1) Initiation of re-evaluating TCM injection program (2) Guidelines for safety evaluation of TCM injection
6	Developing evaluation standards for classic TCM formula	(1) Formulate methods and standards of pharmacy and biology (2) The development of quality markers	Guidelines for application to the classic TCM formula preparation and substance benchmark
7	Harnessing diverse data to improve pharmacovigilance of TCMs	Monitoring of drug safety	(1) Establishment of China Hospital Pharmacovigilance System (2) Establishment of Database of National Center for ADRs Monitoring
8	Evaluating the value of integrative medicine in clinical practice with scientific research	Research evidence on the efficacy of integrative medicine	Developing guidelines and funding for clinical practice of integrative medicine
9	Advancing the regulatory capacity to encourage innovation in TCMs	(1) Technologies of drug discovery (2) Demand for the assessment of new products	(1) Develop biochemical techniques and cutting-edge technologies for drug discovery (2) Equip new tools, methodologies and techniques for the assessment of new products
10	Advancing a vision of collaboration for RS development in TCMs	The challenge of drug innovation development requires to cooperate with all stakeholders	(1) Set up scientific research bases or centers (2) International scientific collaboration

**Fig. 1:**
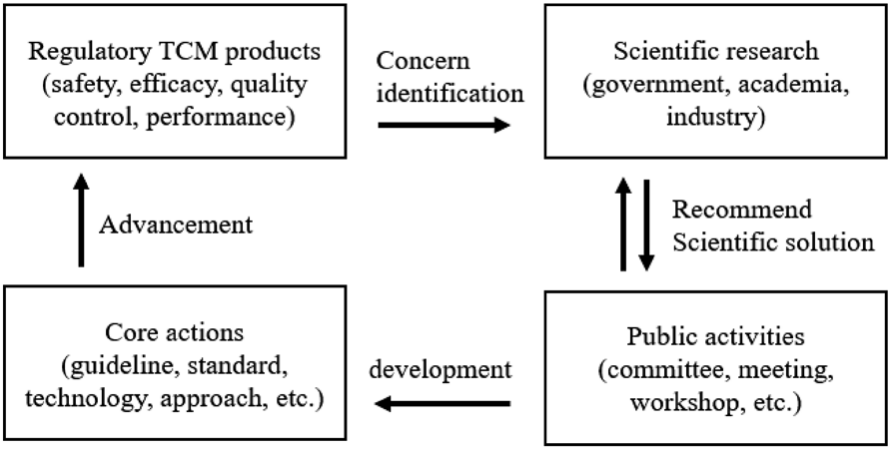
The process of advancing RS for TCMs in China

Figure 1 summarizes an overview of the process of advancing RS for TCMs in this study. When the evaluation tools and concepts for medical innovation products fall short, NMPA should work proactively with manufacturers and the scientific community to identify and resolve critical development concerns and stimulate research through some activities, and then makes this information available to the public through core action to advancing the development of regulatory medical products. It is also important for the NMPA to constantly adjust and adopt new ideas and strategies in TCMs regulation to ensure quality and safety, and to promote continuous innovation in scientific research and development of TCM products, forging the collaborations with all stakeholders to tackle any technical concerns.

In light of the current challenges in the scientific development of TCMs, as Liu et al. proposed [147], a top-level design, and a top-down approach is needed for the sustainable development of RS in TCMs in China. Two principles unique to TCMs must be taken into consideration: (1) the development of TCMs should be considered as a “reverse transformation” of “clinical-trial-clinical” (clinical traditional use experience - clinical trial - clinical use); and (2) the research design of the clinical efficacy re-evaluation should be guided according to the principle of evidence-based medicine. In the process of implementing actions for RS development in TCMs, close attention should be paid to the development of policies, regulations, technical guidelines, technical methods and technical standards of TCMs research and development to effectively resolve the influencing factors affecting the quality, safety and efficacy of TCMs. It is also necessary to strengthen the cooperation among drug regulatory authorities, academia and industry, and improve the RS capacity of drug regulators so as to truly develop and utilize the scientific and technological advancements to promote the progress of RS in TCMs.

## Limitations

There are some limitations in this paper. Firstly, considering that developing and adopting regulatory science especially in TCMs is still at its infancy, the breadth and depth of information available is limited. Therefore, instead of using systematic review suitable for analyzing exhaustive amount of information, the approach of scoping review was employed to capture key findings relevant to the research scope as much as possible. Secondly, the 10 priority areas in this paper are insufficient to fully identify and explain the possible variation of the current RS development in traditional medicines around the world. However, the paper findings may be used to inform the implementation strategy in the priority areas and summarize core actions to promote the development and address the current concerns of TCMs regulation in China and abroad. As outlined in the action plan, TCMs are considered one of the RS priorities calling for immediate actions to accelerate advancement.

## Conclusions

RS for TCMs in China encompasses revolution of operational procedures, advancement in science and technology, and cross-boundary collaborations. Such experiences should be used to facilitate communications among drug regulatory authorities to promote standardized and scientific regulation of traditional medicines.

## Data Availability

Data sharing is not applicable to this article as no datasets were generated or analyzed during the current study.

## Abbreviations

RS: Regulatory science
NMPA: The National Medical Products Administration
TCMs: Ttraditional Chinese medicines
TCM: Traditional Chinese Medicine
FDA: The Food and Drug Administration
EMA: European Medicines Agency
PMDA: Pharmaceuticals and Medical Devices Agency
DRAs: Drug regulatory authorities
MAH: Marketing authorization holder
QbD: Quality by Design
ICH: The International Conference on Harmonisation of Technical Requirements for Registration of Pharmaceuticals for Human Use
Ch.P: Chinese Pharmacopoeia
PAT: Process analysis technology
RCTs: Randomized controlled trials
RCT: Randomized controlled trial
CTCMF: Classic TCM formula
GEP: Good engineering practice
GAP: Good Agriculture Practice
PV: Pharmacovigilance
ADE: Adverse drug event
ADR: Adverse drug reaction
ADRs: Adverse drug reactions
ENCePP: European Network of Centres for Pharmacoepidemiology and Pharmacovigilance
IM: Integrative medicine
ICMRA: International Coalition of Medicines Regulatory Authorities
PIC/S: Pharmaceutical Inspection Convention and Pharmaceutical Inspection Co-operation Scheme
GCRSR: Global Coalition for Regulatory Science Research
GRP: Good Regulatory Practices
IMDRF: International Medical Device Regulators Forum
